# Differential Performance and Parasitism of Caterpillars on Maize Inbred Lines with Distinctly Different Herbivore-Induced Volatile Emissions

**DOI:** 10.1371/journal.pone.0047589

**Published:** 2012-10-24

**Authors:** Thomas Degen, Nenad Bakalovic, David Bergvinson, Ted C. J. Turlings

**Affiliations:** 1 Laboratory of Fundamental and Applied Research in Chemical Ecology, Institute of Biology, University of Neuchâtel, Neuchâtel, Switzerland; 2 Centro International de Mejoramiento de Maíz y Trigo (CIMMYT), Texcoco, Edo. de México, México; Centro de Investigación y de Estudios Avanzados, Mexico

## Abstract

Plant volatiles induced by insect feeding are known to attract natural enemies of the herbivores. Six maize inbred lines that showed distinctly different patterns of volatile emission in laboratory assays were planted in randomized plots in the Central Mexican Highlands to test their ability to recruit parasitic wasps under field conditions. The plants were artificially infested with neonate larvae of the fall armyworm *Spodoptera frugiperda*, and two of its main endoparasitoids, *Campoletis sonorensis* and *Cotesia marginiventris*, were released in the plots. Volatiles were collected from equally treated reference plants in the neighbourhood of the experimental field. The cumulative amount of 36 quantified volatile compounds determined for each line was in good accordance with findings from the laboratory; there was an almost 15-fold difference in total emission between the two extreme lines. We found significant differences among the lines with respect to the numbers of armyworms recovered from the plants, their average weight gain and parasitism rates. Average weight of the caterpillars was negatively correlated with the average total amount of volatiles released by the six inbred lines. However, neither total volatile emission nor any specific single compound within the blend could explain the differential parasitism rates among the lines, with the possible exception of (*E*)-2-hexenal for *Campoletis sonorensis* and methyl salicylate for *Cotesia marginiventris*. Herbivore-induced plant volatiles and/or correlates thereof contribute to reducing insect damage of maize plants through direct plant defence and enhanced attraction of parasitoids, alleged indirect defence. The potential to exploit these volatiles for pest control deserves to be further evaluated.

## Introduction

The emission of volatile organic compounds by plants as a response to herbivore attack is a widespread phenomenon. Such herbivore-induced volatile emissions have commonly been interpreted as a means of indirect defence of the plant, since predators and parasitoids can use these odorous cues to locate their herbivorous prey and hosts, respectively [1], [2]. Most studies on the function of herbivore-induced plant volatiles (HIPVs) are laboratory based and only a few have demonstrated a HIPV-mediated increase in carnivore attack rates on herbivores under field conditions [3]–[5]. The limited evidence that plants actually benefit from attracting natural enemies of herbivores continues to raise scepticism as to the indirect defence function of inducible volatiles, especially in the case of attracting parasitoids [6]–[11].

The tritrophic study system comprising maize, caterpillars and associated parasitoids represents one of the first examples demonstrating the importance of HIPVs in host location by parasitic wasps [12], but so far the phenomenon has not been studied thoroughly under field conditions for the said group of organisms. Somewhat equivocal circumstantial evidence for the action of HIPVs in the field stems from a study carried out in Switzerland: more entomophagous insects - parasitic Hymenoptera, Anthocoridae and Syrphidae - were caught with sticky traps near maize plants that were mechanically damaged and treated with caterpillar regurgitant than near healthy plants [13]. Dispensing synthetic green leaf volatiles in maize fields had little effect on the attraction of beneficial insects [14]. In another study conducted at the same subtropical field site in Mexico, treating maize seedlings with two elicitors, methyl jasmonate and benzo-(1,2,3)-thiadiazole-7-carbothioic acid S-methyl ester (BTH), likewise only marginally affected parasitism rates [15]. Covering different tritrophic systems, several other field studies aimed at supporting a prime assumption of indirect defence by testing whether volatile compounds indeed attract carnivorous arthropods in the field. To this end, plants were either artificially manipulated to induce emission of HIPVs, e.g. by spraying them with jasmonic acid [5], [16], or synthetic formulations of single volatile compounds or mixtures thereof with putative attractive activity were released from dispensers [3], [17]. Only few studies have tested whether the variation in HIPV-release naturally manifested in plant accessions is reflected by differential predation or parasitism in the field. Poelman *et al.* [18] found that cultivars of *Brassica oleracea* identified as most attractive to parasitoids in the laboratory also sustained highest proportions of parasitism in the field and they attributed this finding to intraspecific variation in HIPVs. Other field evidence stems from a belowground interaction: a maize variety releasing an attractant for entomopathogenic nematodes from its roots when attacked by the coleopteran pest *Diabrotica virgifera* exhibited a fivefold higher nematode infection rate of the beetle larvae than a variety that does not produce this signal [19]. The importance of the attractant, the sesquiterpene (*E*)-β-caryophyllene, was further confirmed with a transgenic approach that restored signal production in a maize line that had lost it [20].

In the current study, we relied completely on existing intraspecific variation to clarify whether differential release of aboveground HIPVs by maize plants *Zea mays* in the field leads to differential parasitism of caterpillars. Earlier laboratory studies had revealed extensive genetic variation among maize hybrids [21] and maize inbred lines [22], with regard to both the total amount of volatiles emitted and the relative proportions of individual compounds present in the odour blend. Out of 31 previously screened inbred lines [22] we selected six, comprising three “low emitters” and three “high emitters” in terms of total volatile emissions. These lines were planted in small replicated plots in the altiplano of central Mexico to test the hypothesis that higher HIPV release would lead to higher parasitism rates. For this, we artificially infested the plants with neonate larvae of the fall armyworm *Spodoptera frugiperda* (Smith) (Lepidoptera: Noctuidae), and released two species of parasitoids inside the plots, namely *Campoletis sonorensis* (Cameron) (Hymenoptera: Ichneumonidae) and *Cotesia marginiventris* (Cresson) (Hymenoptera: Braconidae).

The fall armyworm counts amongst the most destructive insect pests of maize in the Americas [23]. It has been proposed that enhancing herbivore-induced volatile emissions in crop plants may help to increase the effectiveness of natural enemies in locating their prey, which eventually is supposed to result in reduced yield losses [24], [25]. One of the prerequisites inherent to such a strategy seems to be met with our tritrophic system: parasitization by *C. marginiventris* and *C. sonorensis* reduces feeding and weight gain in *Spodoptera littoralis* caterpillars, and as a consequence, young maize plants attacked by a single parasitized larva suffer less feeding damage and, at maturity, produce more seed than plants that are attacked by an unparasitized larva [26]. The present trial provides much needed field evidence for the role of plant volatiles in host location by parasitoids, and represents a first step towards evaluating the practicability of a pest control method based on improved attraction of beneficial insects.

## Materials and Methods

### Field Site and Experimental Design

The field study was conducted at the International Maize and Wheat Improvement Center CIMMYT near Mexico City. The high altitude of 2200 m above sea level allowed us to grow a set of inbred lines from temperate regions of Europe and North America, which were previously shown to differ largely in herbivore-induced odour emissions [22], but were not apt for being tested in the subtropical environment of the lowlands. Furthermore, we could control for fall armyworm infestation, as no significant population of this herbivore existed at this particular location. The incidence of high natural infestation was known to confound results from similar experiments at a subtropical field site (Maria Hoballah Fritzsche, unpublished data). Notwithstanding, we could study the insects near their natural environment, since *S. frugiperda* was observed at low densities on teosinte *Zea mays mexicana* at two locations about 1.5 km and 7 km away from the study field (E. de Lange and T. Degen, unpublished data). In the same survey, only *C. sonorensis* was recorded in the region, parasitizing around 30% of the collected caterpillars.

**Figure 1:**
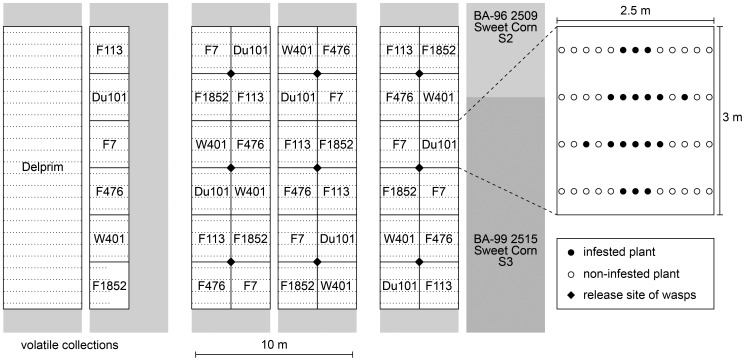
Arrangement of the experimental plots with the 6 maize inbred lines. The enlargement shows the generalized location of the plants that were infested with *Spodoptera frugiperda* larvae inside the plot, the actual pattern being somewhat variable among the plots depending on the availability of suitable plants. The six plots to the left and the bigger plot with plants of the variety “Delprim” were used for volatile collections.

In order to minimize potential effects of position and soil gradients, the plots, 2.5 m×3 m in size, with six replicates per line were arranged in a Latin square design with the restriction that each “high emitter” was neighbouring a “low emitter” (Fig. 1). A border planted with sweet maize surrounded the experimental plots. An additional separate plot for each line contained plants that were used for odour collections. In a neighbouring bigger plot, plants of the model hybrid Delprim were grown for comparative purposes, i.e. to assess larval mortality and odour release of infested and non-infested plants over time.

### Plants

The six inbred lines chosen for the experiments represented the extremes in terms of total volatile emissions under laboratory conditions: the lines W401, F7 and F113 release only very small amounts of volatiles after induction, whereas F1852, F476 and Du101 consistently emit large quantities [22]. By tendency, this difference holds also true for the total quantity of constitutively emitted volatile compounds such as myrcene and linalool, i.e. lines with high constitutive odour production tended also to release higher amounts of induced odours [22]. On 7 May 2001, 52 seeds were sown in each plot. Germination rates were lower for two lines, leading to lower plant densities in the respective plots: on 3 June 2001, just before the start of the experiments, plots of F1852 and F476 consisted on average of 36 and 42 seedlings, respectively, as compared to 51–52 seedlings for the other four inbred lines. An additional plot was planted on 29 May 2001 with our long-time laboratory standard, the hybrid Delprim.

### Insects

The fall armyworm larvae *S. frugiperda* that were used to infest the plants were obtained from a permanent laboratory culture kept at the CIMMYT. The parasitoids released in the field belonged to two species, the ichneumonid *Campoletis sonorensis* and the braconid *Cotesia marginiventris*. They originated from laboratory cultures that were started from parasitized *S. frugiperda* larvae of different stages collected on February 15 and 16, 2001, from heavily infested maize fields at two locations near today’s Ernest W. Sprague experimental station in Agua Fria (Puebla, Mexico) [27]. The wasps were reared on fall armyworm larvae fed with artificial diet. It should be noted that for the volatile collections in the laboratory we used larvae of *Spodoptera littoralis* reared from eggs that were supplied by the Syngenta rearing facilities (Stein, Switzerland).

### Infestation of Plants with Neonate *Spodoptera Frugiperda* Larvae

**Figure 2:**
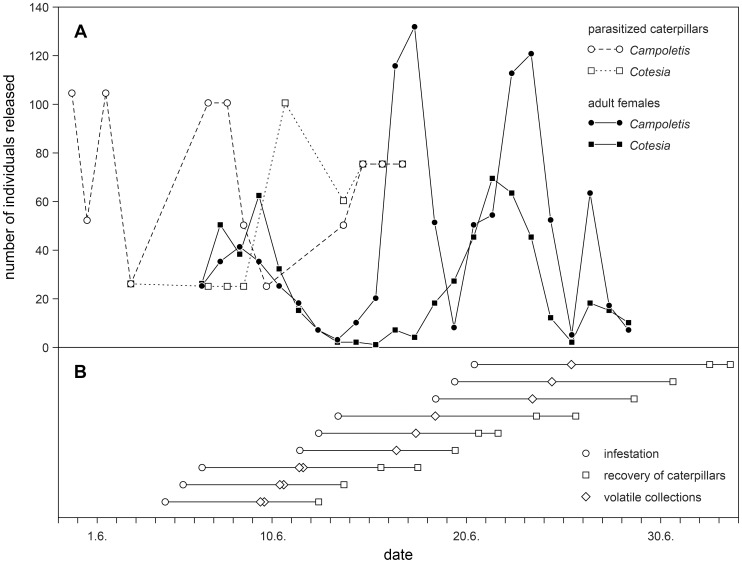
Release of parasitoids and infestation of plants with caterpillars. A) Numbers of female wasps and parasitized caterpillars released over the experimental period. B) Timing of infestation with neonate *Spodoptera frugiperda* larvae and of the subsequent recovery of the caterpillars remaining on the infested plants as well as of the volatile collections, whereby each line represents groups of plants that were infested together.

A mechanical dispenser for lepidopteran larvae, the so-called “bazooka”, was used to inoculate the plants [28]. This infestation device can be pre-calibrated to deliver a more or less uniform number of neonate larvae mixed with corncob grits into the whorl of the maize plants. The average number of larvae counted with test shots in the laboratory was used to determine the number of shots required to target the total number of larvae applied per plant in the field. The infestations were carried out on nine different days (Fig. 2B), usually between 9 am and 11 am. Successive infestations instead of a collective treatment of all plants on a single day were necessary for practical reasons, i.e. because of limited availability of insects and for spreading out the workload. The coefficients of variation calculated from eight test shots on each infestation day ranged from 14 to 42%, but for the total quantity of larvae released on individual plants on the same day, the coefficient of variation is expected to be lower, around 10 to 20%, since depending on the day 2–5 shots were applied per plant. The mean number of caterpillars deposited on the plants as estimated from the test shots was somewhat variable among the days and spanned from 59 to 91 individuals (75±12, mean ± std. dev.), which falls well within the range of egg masses laid on a single host plant that can be observed under natural conditions [29], [30]. All lines were treated according to the same time schedule in order to avoid any bias caused by variations in the interval between release and recovery of caterpillars.

### Release of Parasitoids

The evening of the day that wasps emerged from their cocoons they were sexed and transferred to 1-litre plastic bottles (Joy Tecnica Plastica, S.A. Mexico; www.joytp.com). The following morning these containers, attached to small wooden sticks, were positioned at nine points inside the experimental field (Fig. 1). The lids of the bottles were then opened so that the wasps, which before did not have any access to plants, could fly off spontaneously. In this way, a total of 4470 naïve wasps were released over the whole experimental period: 1004 female together with 1772 male *C. sonorensis*, as well as 570 female with 1124 male *C. marginiventris* (Fig. 2A). In parallel, 886 *S. frugiperda* larvae parasitized by *C. sonorensis* and 486 larvae parasitized by *C. marginiventri*s were placed onto maize plants in the border surrounding the experimental field.

### Assessment of Performance and Parasitism

Out of 648 infested maize plants a total of 540 were eventually checked for the presence of herbivores and the accompanying parasitoids. Eight to thirteen days after being infested (Fig. 2B), the plants were excised at the base, placed into paper bags, which were sealed with a stapler to prevent insects from escaping, and transferred to the laboratory. Within the next two days, each plant was carefully inspected to find and collect the caterpillars. The presence of other arthropods, herbivores and/or predators was also noted. The *Spodoptera* larvae collected from a single plant were counted and weighed together on an analytical balance to assess their total biomass. Then they were placed singly in the compartments of Elisa plates (Nunc and Falcon multidish plates with 24 wells) containing a piece of 1–1.5 g artificial diet and they were reared out until pupation. A piece of paper towel was folded twice and put between the plate and the lid to prevent the larvae from escaping. The plates were incubated in a controlled environment room at 27°C and 70%–100% r. h., and were checked for the presence of parasitoids on up to three occasions. Whenever necessary, fresh diet was added to the wells.

For comparative purposes, larval performance of *S. frugiperda* was also assessed under laboratory conditions. To this end, ten second instar *S. frugiperda* larvae were placed on about 15-day old maize seedlings (n = 12 for each line) and left feeding for one day before being transferred onto another plant of the same line for another one-day period. To test for a potential effect of induced resistance, half of the plants was injected with 3×10 µl of caterpillar regurgitant [22] about 1 day before and again about 1.5–0.5 h before the plants were infested, an induction method that allowed for equal treatment of all the plants, avoiding potential effects of differential larval feeding. The larvae were weighed together on days 1, 2 and 3. As a measure for growth rate we used the slope of the regression of time on the three log-transformed average weights.

### Shoot Dry Weight and Leaf Toughness

After being inspected for the presence of insects, each disassembled plant collected from the experimental field plots was put back into a paper bag, heated in an oven, and subsequently weighed. As a measure of leaf toughness, an important factor contributing to maize resistance [31], the force in g/mm^2^ required to puncture a random sample of freshly excised leaves of the six maize inbred lines was determined at the end of the experiment with a rounded cylindrical probe (of 1 mm diameter) attached to an Instron device (Instron Engineering Corporation).

### Volatile Collections

Five days after infestation with neonate *S. frugiperda* larvae, volatiles were collected from individual plants between 9 am and 11 am and, occasionally, on the three days of the first week also between 2 pm and 4 pm (Fig. 2B). Transparent polyethylene terephthalate (PET) bags were prepared from a Nalophan® NA 20 roll (Kalle, Germany; 20 µm thickness; 30 cm diameter) by cutting them into 60 cm or 80 cm long pieces (depending on plant size). These plastic “sleeves” were placed over selected maize plants, the lower part was clamped around the stem below the first leaf, and the upper part was closed above the plant with wire, just prior to the start of air sampling. Battery-powered portable Buck I.H. pumps (A.P. Buck, Inc., Orlando FL) were used to pump air in and out of the bags over a closed loop. Air from the outlet of the pump passed via a plastic tube into a charcoal filter that purified the air before it entered the bag from below. Volatile compounds that accumulated inside the bag were trapped by pulling air through a filter that was inserted in the upper part of the sleeve. The trap consisted of a glass tube (4 mm ID) packed with 25 mg Super-Q polymer (80–100 mesh) (Alltech, Deerfield, Illinois, USA) that was connected via another plastic tube to the inlet of the pump. The charcoal filter and the Super-Q trap were inserted into the bag through glass tubes fitted with a screw cap containing an open Teflon-coated septum. The pumps were calibrated in such a way as to attain an airflow of 0.8 litre/min. After trapping the volatiles for 2 h, the Super-Q filters were extracted on the same day with 150 µl dichloromethane (HPLC grade), and 200 ng of n-octane and n-nonyl acetate (Sigma, Switzerland) in 10 µl dichloromethane were added to the samples as internal standards. The solutions were kept in 2 ml-vials (Infochroma AG, Zug, Switzerland) and, with exception of periods of transportation, stored at about −80°C until analysis.

For comparative purposes, volatiles of the six inbred lines were also collected in the laboratory for a duration of 4 h following the method described by Turlings et al. [32]. To this end, 9–12 day old seedlings of the six inbred lines were infested with 10 second-instar larvae of *Spodoptera littoralis*. Volatiles were collected 15 h after infestation, with six replicates per inbred line.

### Chemical Analysis: Identification and Quantification

Aliquots of 2 µl of each sample were injected into a gas chromatograph (Hewlett Packard HP 6890) coupled to a mass spectrometer operated in electron impact mode (Hewlett Packard HP 5973 Network Mass Selective Detector; transfer line 230°C, source 230°C, ionisation potential 70 eV) in the pulsed splitless mode onto a non-polar column (HP-1 MS, 30 m, 0.25 mm ID, 0.25 µm film thickness, Alltech Associates, Inc., USA) with Helium as carrier gas at constant flow (0.9 ml/min). After injection, the temperature was maintained at 40°C for 1 min, then increased to 250°C at 8°C/min followed by a post-run of 30 min at 250°C. The volatiles were identified by comparing their mass spectra with those of the NIST05 library and by comparing their retention times with those of previous analyses. For some compounds no authentic standards were available. Hence, their identification is based solely on their mass spectra and must be considered tentative (Table 1).

**Table 1:** Average quantities (in ng; mean ± s.e; n = 12) of volatiles compounds collected in the field from the headspace of infested maize plants belonging to six different inbred lines.

volatilecompound		identification	F7	W401	F113	F1852	Du101	F476	*r*	*p*
1	(*Z*)-3-hexenal	RT, MS	31.9±11.3	6.4±2.2	12.4±3.2	9.4±2.2	28.6±15.2	14.1±3.8	−0.20	0.709
2	(*E*)-2-hexenal	RT, MS	13.7±7.4	1.9±1.4	5.6±2.7	1.8±1.2	10.4±4.8	2.8±1.6	−0.12	0.821
3	(*Z*)-3-hexen-1-ol	RT, MS	72.8±24.9	71.2±21.4	66.9±14.9	83.7±21.4	103.6±35.3	113.5±22.5	0.19	0.712
4	α-pinene	RT, MS	6.5±2.8	13.0±4.7	5.1±1.1	12.8±2.3	10.7±4.0	3.2±0.9		
5	β-myrcene	RT, MS	15.6±2.1	13.2±1.6	41.0±4.6	13.7±2.4	19.3±1.4	142.9±10.4	0.46	0.365
6	(*Z*)-3-hexenyl acetate	RT, MS	62.8±16.2	110.7±31.1	297.1±70.5		113.1±36.3	282.5±55.2	**0.96**	**0.003**
7	hexyl acetate	RT, MS	8.8±2.0	22.0±5.0	40.1±7.3		10.0±1.9	44.2±9.8		
8	limonene	RT, MS	4.6±1.4	28.3±4.1	17.7±1.0	52.6±7.6	42.6±3.9	12.8±1.6		
9	ocimene	RT, MS		2.6±1.0	0.5±0.4	2.6±1.0	5.4±1.2	3.1±0.7	**0.90**	**0.016**
10	linalool	RT, MS	50.6±8.3	6.4±2.4	62.2±10.2	15.3±3.5	215.2±26.9	1596.3±199.4	0.56	0.247
11	(*E*)-4,8-dimethyl-1,3,7-nonatriene	RT, MS	36.5±5.8	13.8±6.3	11.4±2.0	39.8±6.3	165.9±25.5	170.2±21.4	**0.89**	**0.016**
12	methyl salicylate	RT, MS	34.8±10.7	63.5±18.1	32.7±10.6	30.3±11.6	34.3±14.2	39.4±17.7	−0.04	0.938
13	phenethyl acetate	RT, MS	0.1±0.1	2.4±0.6	7.0±1.8		3.4±0.6	11.9±2.5	**0.87**	**0.025**
14	indole	RT, MS	1.0±0.8	0.6±0.3	2.0±0.9	1.8±1.2	28.6±6.9	89.5±19.2	0.68	0.141
15	methyl anthranilate	RT, MS		1.2±0.5	0.0±0.0	5.9±0.7	12.6±1.3	1.9±0.6	**0.91**	**0.013**
16	geranyl acetate	RT, MS	0.8±0.2		8.0±1.4			70.6±11.5	**0.82**	**0.045**
17	cycloisosativene	MS		4.1±0.4	4.1±0.6	141.9±10.7		123.0±9.8	**0.94**	**0.005**
18	α-ylangene	MS				119.0±10.8		114.8±9.3		
19	α-copaene	RT, MS	0.9±0.2		45.5±4.0	27.0±2.7	71.6±7.0	68.2±5.7	**0.89**	**0.017**
20	unknown sesquiterpene 1(β-bourbonene)	MS	2.6±0.6		10.2±1.3	23.4±1.9	53.1±5.9	71.9±7.1		
21	unknown sesquiterpene2 (zingiberene-type)	MS		0.7±0.7	0.7±0.4	48.6±8.4	29.9±3.0	135.0±15.8		
22	unknown sesquiterpene3 (zingiberene-type)	MS				637.8±129.6				
23	unknown sesquiterpene4 (isocaryophyllene-type)	MS	11.7±1.9	0.2±0.2			73.6±7.9	187.9±22.4		
24	unknown sesquiterpene5 (bergamotene-type)	MS				57.3±10.9		0.8±0.6		
25	β-caryophyllene	RT, MS	188.5±32.0	25.0±5.1	18.4±2.9	65.2±22.9	2254.2±252.5	4607.8±596.6	**0.97**	**0.001**
26	(*E*)-α-bergamotene	RT, MS	6.3±1.1	48.6±8.6	44.0±9.6	80.5±17.6	247.3±40.5	121.5±16.1	**0.91**	**0.012**
27	unknown sesquiterpene 6	MS	0.1±0.1	7.7±1.0	3.5±1.3	13.4±1.7	20.1±3.2	21.9±2.7	**0.94**	**0.005**
28	(*E*)-β-farnesene	RT, MS	13.4±3.0	163.1±30.4	119.1±29.9	178.2±32.1	638.2±93.4	116.9±13.3	**0.93**	**0.007**
29	α-humulene	RT, MS	14.7±2.2	8.6±0.8	4.0±0.5	5.2±1.3	120.7±14.3	267.0±29.7	**0.94**	**0.005**
30	germacrene D	RT, MS	0.2±0.2	3.5±0.3	9.1±0.6	36.0±2.6	64.8±8.5	106.2±9.3	**0.96**	**0.002**
31	unknown sesquiterpene 7 (β-selinene)	MS	8.6±1.2		261.6±63.3			24.9±6.1		
32	unknown sesquiterpene 8	MS	3.9±1.1	0.6±0.3	267.4±62.7		3.9±1.2			
33	unknown sesquiterpene 9 (β-cadinene)	MS		1.6±0.5				206.3±21.6	−0.40	0.435
34	unknown sesquiterpene 10 (β-bisabolene)	MS			10.6±4.6	120.7±18.9	7.4±3.1		**0.95**	**0.004**
35	unknown sesquiterpene 11 (δ-cadinene)	MS	4.2±0.4	3.8±0.5	15.7±1.4	40.0±3.9	37.0±3.9	50.5±3.9	0.26	0.619
36	(*E*,*E*)-4,8,12-trimethyl-1,3,7,11-tridecatetraene	RT, MS	8.3±1.2	36.3±5.5	26.9±3.0	32.8±4.7	74.6±7.0	65.7±6.7	0.58	0.225
	sum of all compounds		604.0±86.5	661.2±91.3	1450.6±153.2	1896.8±246.8	4500.2±438.2	8889.4±739.0	**0.92**	**0.009**

The compounds are arranged in order of increasing retention time. Identifications based on mass spectra (MS) alone must be considered tentative. When an authentic standard was available, retention time (RT) served as additional criterion. For comparative purposes, coefficients *r* (Pearson product moment correlations) and the respective probabilities *p* are given for correlations with average amounts of volatiles (n = 6) obtained from collections carried out in a six-arm olfactometer (means of log-transformed values).

For quantification we injected another 3 µl aliquot of each sample into a Hewlett-Packard HP 6850 series gas chromatograph (Palo Alto, CA) equipped with an automated on-column injection system and a flame ionization detector, using comparable parameters, i.e. the same kind of apolar column (HP-1) and an identical temperature program. The quantities of 36 volatile compounds were calculated by comparing their peak area with those of the internal standards, n-octane for the first 9 eluting compounds and n-nonyl acetate for the remaining compounds listed in Table 1.

### Statistics

Since several of the assessed parameters were not normally distributed, we used the non-parametric Kruskal-Wallis test to check for differences among the lines. For parametric multifactorial analyses (ANOVA, ANCOVA) data were log- or arcsin-transformed when necessary in order to reduce heteroscedasticity and to approach normal distribution. The average values of the parameters for each line were used to compute a correlation matrix. Principal component analyses were carried out with the Canoco software (Plant Research International, Wageningen, the Netherlands) and were based on log-transformed values of the parameters, e.g. the amounts of volatiles.

## Results

### Herbivore Survival, Performance and Dispersal

**Figure 3:**
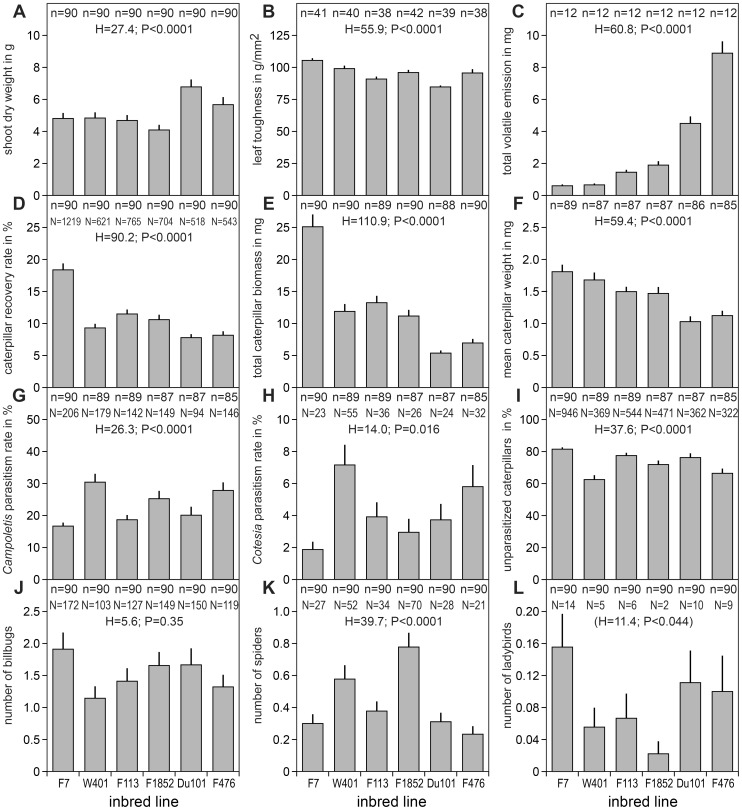
Overview of the parameters assessed for six maize inbred lines arranged in order of increasing total volatile emission. A-C) plant parameters; D-F) performance of the herbivore *Spodoptera frugiperda*; G-I) parasitism; J-L) natural infestation with herbivores and potential predators; n = number of plants; N = number of arthropods.

A total of 4648 *S. frugiperda* caterpillars, as well as 22 *C. sonorensis* cocoons and 1 *C. marginiventris* cocoon were directly collected from the plants. About 6% of the caterpillars were noticeably larger in size than their congeners collected from the same plant (Table 2), 14.7% and 6.8% in week 2 and 3, respectively, but only 0.04% in week 1 (see Fig. 2B for the schedule). Their average weight was about 26 times higher. We conclude therefore that these larger caterpillars originated from the earlier infestations in week 1 and 2, respectively, and had migrated onto plants treated in week 2 and 3. Such dispersal from one plant to another is a known phenomenon for neonate fall armyworm larvae [29], [33]. To avoid any bias, these older individuals were excluded from the calculation of performance parameters. The almost complete absence of such larvae on plants infested in the first week – only 1 out of 2301 - implies that there was no significant natural *S. frugiperda* infestation. Yet, we also recorded a total of 31 caterpillars belonging to other lepidopteran species and a total of 820 billbugs (Coleoptera: Curculionidae), which thereby represented the most important other type of herbivorous insects on the plants (see also Fig. 3J).

**Table 2:** Overview of the numbers of insects and their status after collection (begin) and after rearing out (end).

time	species	status and stage	N
begin	*Spodoptera frugiperda*	larval instar 1–4	4370
		larval instar 3–6^a^	278
	*Campoletis sonorensis*	coocon^a^	22
	*Cotesia marginiventris*	cocoon^a^	1
end	*Spodoptera frugiperda*	missing (e.g. empty wells)^b^	89
		dead larval instar 1–4^b^	456
		dead or alive larval instar 5–6^c^	162
		dead or alive prepupa andpupa^c^	2310
		Spodoptera adult^c^	542
	*Campoletis sonorensis*	unknown	5
		dead larva	210
		cocoon	166
		adult wasp	535
	*Cotesia marginiventris*	unknown	1
		dead larva	80
		cocoon	24
		adult wasp	91

a originating from earlier infestations of other plants or from natural infestation as judged from size comparison with the other caterpillars; excluded from assessment of performance parameters, i.e. recovery rate and biomass.

b excluded from the calculation of parasitism rates.

c included in the calculation of parasitism rates as unparasitized.

The proportion of caterpillars remaining on the monitoring plants of the variety Delprim, which had been infested with about 70 neonates, quickly dropped to below 50% after four days and then levelled off to reach about 10% on day 20. Recovery rates differed significantly among the inbred lines (Kruskal-Wallis test: df = 5, *H* = 90.2; *p*<0.0001) (Fig. 3D). According to an ANCOVA, recovery rate (arcsin-transformed) not only depended on the line (*F*_5, 525_ = 43.0, *p*<0.0001), but also varied with the date of infestation (*F*_8, 525_ = 59.1, *p*<0.0001), and - as expected - decreased with increasing duration from infestation until recovering the larvae from the plants (*F*_1, 525_ = 10.8, *p* = 0.0011). Suboptimal application of the bazooka device on a few days was responsible for some inconsistencies among the dates.

Since the total biomass of the caterpillars obtained for a given plant necessarily was strongly correlated with the number of caterpillars recaptured (Table 3), these two measurements showed the same tendencies. In fact, the differences among the lines were even more pronounced for caterpillar biomass (Kruskal-Wallis test: df = 5, *H* = 110.9; *p*<0.0001) (Fig. 3E) because caterpillars grown on plants from which more individuals were recovered also tended to be heavier on average (Spearman rank correlation ρ = 0.23, n = 521 *p*<0.0001). As with recovery rate, total biomass (log-transformed) was influenced by the factors line (ANCOVA, *F*_5, 522_ = 52.0, *p*<0.0001) and date of infestation (*F*_5, 522_ = 70.5, *p*<0.0001), but not by the time between infestation and collection (*F*_1, 522_ = 0.037, *p* = 0.85). The average weight of the caterpillars as well differed significantly among the lines (Kruskal-Wallis test: df = 5, *H* = 59.4; *p*<0.0001) (Fig. 3F). The interval from infestation to recollection of caterpillars and subsequent weighing was practically identical for all the lines, therefore differences in average weight can be considered equivalent to differences in growth rate, which in turn is related to developmental time.

**Table 3:** Correlation coefficients *r* (Pearson product moment correlations) between 6 maize inbred lines for the different parameters assessed in the field, i.e. the mean values shown in Figure 3.

parameter (means)	A	B	C	D	E	F	G	H	I	J	K
A shoot dry weight											
B leaf toughness	−0.609										
C total volatile emission	0.588	−0.361									
D caterpillar recovery rate	−0.460	0.722	−0.566								
E total caterpillar biomass	−0.532	0.794	−0.640	**0.982**							
F mean caterpillar weight	−0.756	0.802	**−0.824**	0.763	**0.862**						
G *Campoletis* parasitism rate	−0.106	0.085	0.297	−0.622	−0.504	−0.127					
H *Cotesia* parasitism rate	0.152	−0.099	0.297	−0.660	−0.523	−0.174	**0.835**				
I frequency unparasitized caterpillars	0.040	−0.038	−0.306	0.653	0.526	0.144	**−0.989**	**−0.907**			
J number of bill bugs	0.021	0.157	−0.221	0.648	0.512	0.127	−0.770	**−0.952**	**0.845**		
K number of spiders	−0.654	0.122	−0.501	−0.108	−0.041	0.334	0.408	0.031	−0.320	−0.111	
L number of ladybirds	0.498	0.226	0.165	0.503	0.433	0.008	−0.566	−0.344	0.525	0.507	**−0.830**

Bold numbers indicate a significant correlation (Fisher’s r to z *p*<0.05).

**Figure 4:**
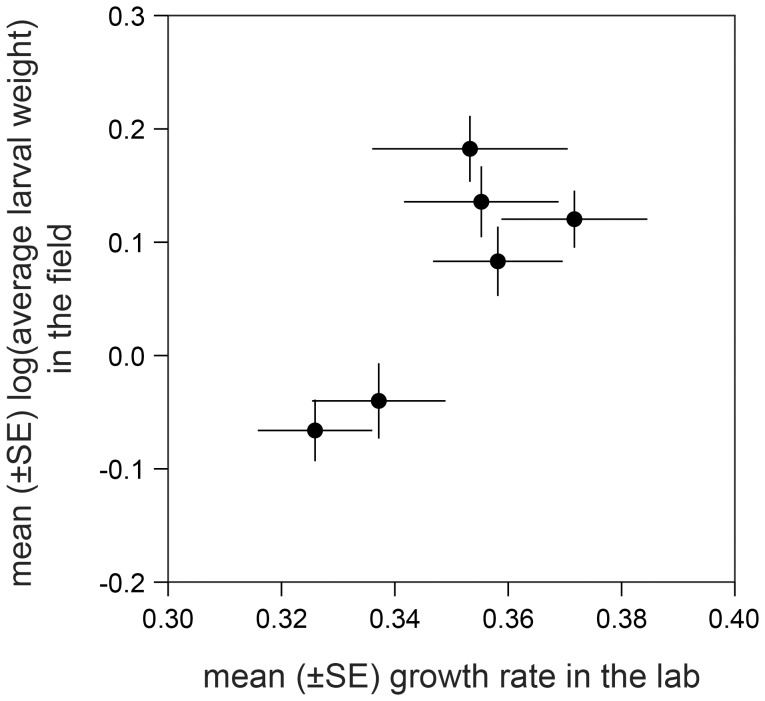
Performance of *Spodoptera frugiperda* caterpillars on the six maize inbred lines under laboratory and field conditions (*r* = 0.81; *p* = 0.0505).

In a laboratory assay meant to test whether induced resistance could be responsible for the differential larval performance observed in the field, growth rates did not differ significantly between plants that were injected beforehand with caterpillar regurgitant and untreated plants (ANOVA *F*_1, 65_ = 0.30, *p* = 0.59) nor among the lines (*F*_5, 65_ = 1.5, *p* = 0.19). Yet, the average larval growth rates on the lines in the laboratory were by tendency in good accordance with the average weights recorded in the field (log-transformed), the correlation being very close to significant (*r* = 0.810, *p* = 0.0505; Fig. 4). It is conceivable that the time during which the larvae could feed on the plants in the laboratory experiment, i.e. three days only as compared to eight to thirteen days in the field, was too short to allow build-up of significant differences in larval weight among the lines.

### Parasitism and Predators

A total of 916 and 196 caterpillars were parasitized by *C. sonorensis* and *C. marginiventris*, respectively. A proportion of 23% and 41%, respectively, of the parasitoids died at the larval stage (Table 2). The larger dead larvae could easily be attributed to *C. sonorensis*, but for some of the smaller larvae species identification might have been slightly less reliable.

As is evident from the total numbers, a significantly higher percentage of caterpillars was parasitized by *C. sonorensis* (23.1%) than by *C. marginiventris* (4.2%) (Wilcoxon Signed Rank Test: *H* = −15.0; *p*<0.0001). Most relevant to the purpose of this study, parasitism rates of the caterpillars significantly varied among the six maize lines. This was the case for *C. sonorensis* (Kruskal-Wallis test: df = 5, *H* = 26.3; *p*<0.0001), as well as for *C. marginiventris* (Kruskal-Wallis test: df = 5, *H* = 14.0; *p* = 0.016), with the differences between the two extreme lines F7 (lowest rate) and W401 (highest rate) being almost two- and four-fold, respectively (Fig. 3G&H). For *C. marginiventris*, parasitism rates tended to increase with the number of caterpillars available on the plants, significantly so in four of the lines (W401, F113, F1852, Du101), the lowest Spearman rank correlation coefficient ρ being 0.159 (F476) and the highest 0.390 (Du101). No such density dependent effect was found for *C. sonorensis*. The most important other carnivorous arthropods found on the sampled plants were spiders (Fig. 3K), followed by ladybirds (Fig. 3L).

### Plant Parameters

The inbred lines differed significantly with respect to shoot dry weight (Kruskal-Wallis test: df = 5, *H* = 27.4; *p*<0.0001; Fig. 3A) and in leaf toughness (Kruskal-Wallis test: df = 5, *H* = 55.9; *p*<0.0001; Fig. 3B). They also showed some morphological variation, e.g. in the number of leaves per g shoot dry weight (Kruskal-Wallis test: df = 5, *H* = 48.4; *p*<0.0001).

Total volatile emission, i.e. the sum of all 36 quantified compounds, was highly different among the lines (Kruskal-Wallis test: df = 5, *H* = 60.8 *p*<0.0001; Fig. 3C), with an almost 15-fold difference between the line with the lowest (F7) and the line with the highest release (F476). Except for the three green leaf volatiles (*Z*)-3-hexenal, (*E*)-2-hexenal, and (*Z*)-3-hexen-1-ol, the Kruskal-Wallis test revealed significant differences among the lines for all compounds (α-pinene, *p* = 0.0025; methyl salicylate, *p* = 0.038; all remaining compounds, *p*<0.0001).

**Figure 5:**
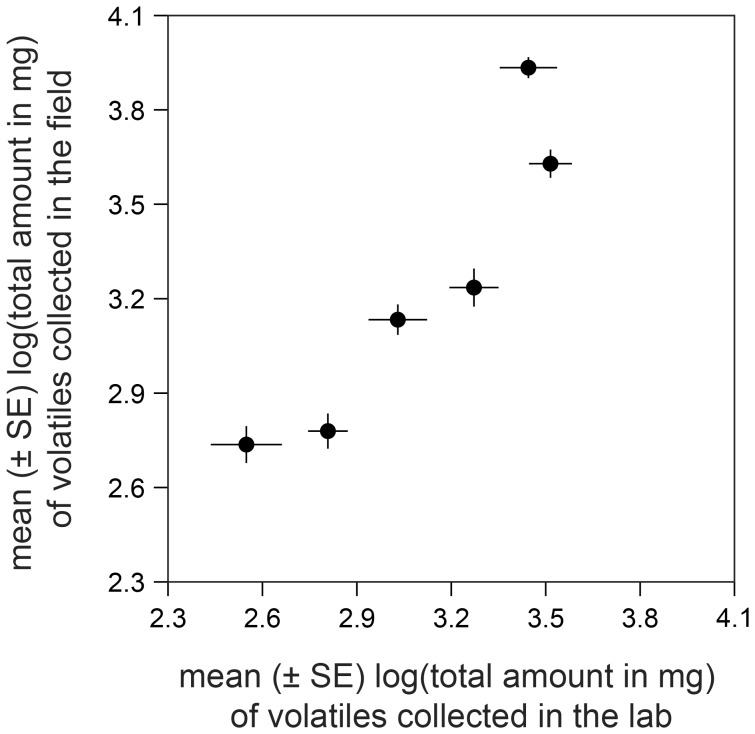
Total volatile emission of the six maize inbred lines according to collections conducted in the laboratory (six-arm olfactometer) as compared to collections carried out in the field (*r* = 0.92, *p* = 0.0093).

The differences in total volatile emission among the six lines reported here from the field agree well with the results from the volatile collections conducted in the laboratory (Fig. 5; for means of log-transformed amounts: *r* = 0.92, *p* = 0.0093, Spearman rank correlation ρ = 0.94, *p* = 0.035) and with the findings of an earlier laboratory study [22] (*r* = 0.91, *p* = 0.012; Spearman rank correlation ρ = 1.0, *p* = 0.025), in both cases obtained by infesting the maize plants with *Spodoptera littoralis* larvae. At the level of individual compounds, there was often a good correspondence as well: for 15 out of 25 compounds found in detectable amounts both in the field and the laboratory the correlation was significant (means of log-transformed amounts; Table 1). While each line retained its specific characteristics, e.g. the complete absence of acetates in F1852, the proportions of individual compounds in the total blend varied considerably between the field and the laboratory for certain compounds. Some were detected in important amounts in the field, whereas they were almost absent in the laboratory, e.g. methyl salicylate in all lines, unknown sesquiterpene 3 in F1852, and unknown sesquiterpenes 7 and 8 in F113 (Table 1). Other volatiles were collected in considerably lower amounts in the field than in the laboratory, such as the three aromatic compounds phenethyl acetate, indole and methyl anthranilate, as well as the two green leafy volatiles (*Z*)-3-hexenal and (*E*)-2-hexenal, but not (*Z*)-3-hexenol.

Volatile collections from four nearby maize plants of the hybrid Delprim infested in the same way as the inbred lines - a total of 13 samples per plant spanning the period from 1 day before to 20 days after infestation – revealed a steady increase in emitted amounts to about four times the initial level from day 1 to day 5 after infestation, after which volatile release started to level off (non-published data Nenad Bakalovic). By contrast, volatile emissions of two non-treated plants did not increase likewise over the same time interval. Yet, in this field experiment, total volatile emission of attacked plants was on average only about 2.5 times higher during the 20-day period than that of non-infested plants, as compared to an about 9-fold increase in the laboratory [22]. This difference might be best explained by the fact that, unlike in the laboratory, in the field untreated plants were not entirely unharmed and suffered attacks by other herbivores and possibly pathogens, leading to odour release above constitutive levels.

Volatile collections on the reference plants where conducted five days after infestation, about halfway through the period during which the larvae were exposed on the plants in the experimental plots (see Fig. 2B). At this time, on average about twice as many caterpillars could be recovered from these reference plants than later from the experimental plants, while their cumulative weight was only about half as big, since the larvae were still much smaller. However, both mean recovery rate (*r* = 0.941, *p* = 0.005) and mean total larval biomass (*r* = 0.859, *p* = 0.028) observed for each line with the plants used for odour collection were significantly correlated with the corresponding mean values recorded for the experimental plants.

### Correlations

Table 3 shows a correlation matrix based on the average values per line of all the variables as presented in Figure 3. Apart from some “inherent” correlations due to mathematical linkage, e.g. between proportions of parasitized and unparasitized caterpillars, there are some correlations that are biologically relevant. The higher the total volatile emission by the reference plants, the poorer was the performance of the *S. frugiperda* caterpillars as expressed by the average weight attained on the respective inbred line. Such significant negative relationships were also found for 9 out of the 36 quantified compounds, namely for the green leaf volatile (*Z*)-3-hexenol, the two homoterpenes (*E*)-4,8-dimethyl-1,3,7-nonatriene and (*E*,*E*)-4,8,12-trimethyl-1,3,7,11-tridecatetraene, as well as for the six sesquiterpenes α-copaene, (*E*)-α-bergamotene, germacrene D, unknown 1, 6 and 11.

Patterns of parasitism rates on the six inbred lines were similar for the two wasp species. In both cases, there was no significant correlation between parasitism rate and total volatile emission of the maize lines. Parasitism rate was significantly correlated with two compounds only, negatively with (*E*)-2-hexenal (*r* = −0.843, *p* = 0.035) in *C. sonorensis* and positively with methyl salicylate (*r* = 0.825, *p* = 0.043) in *C. marginiventris*. Finally, two additional negative correlations were found between the mean number of billbugs and the mean proportion of caterpillars parasitized by *C. marginiventris* as well as between spiders and ladybirds.

**Figure 6:**
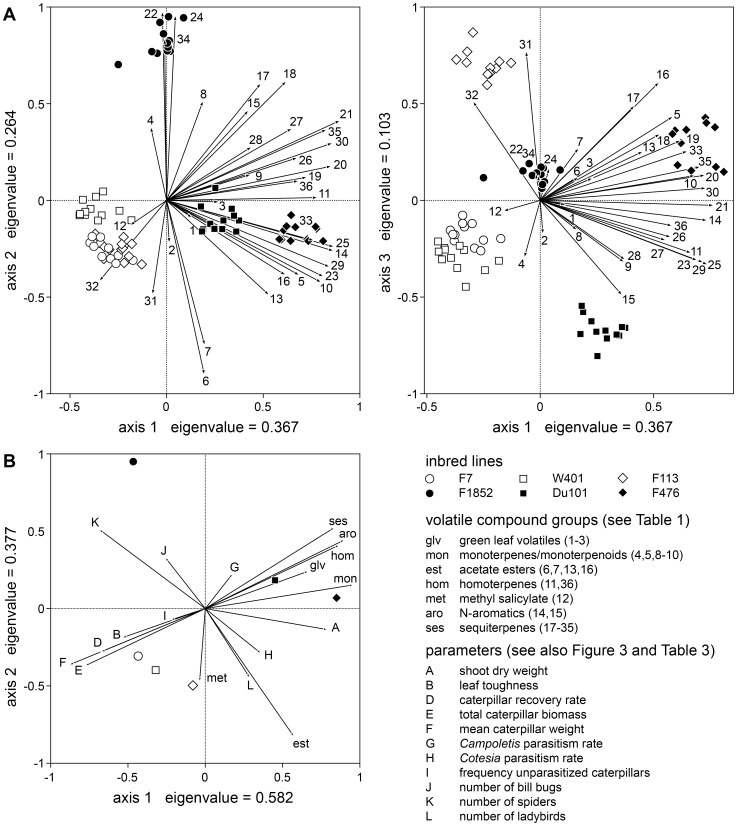
Principal component analysis (PCA) of herbivore-induced volatile emission and of other plant parameters. A) Biplots of a PCA of the 36 volatile compounds (for identity see Table 1) released by the individual maize plants. B) PCA of the average values for each line of the volatiles pooled into seven groups and of the other variates (A, B, D–L as in Fig. 3 and Table 3).

Neither the overall amount of volatiles nor the quantity of every individual compound necessarily represent the information that is used by the animals. That is why we carried out a principal component analysis (PCA) of the volatiles and then searched for correlations of the factor scores with the other parameters listed in Table 3. The first two axes of a PCA on the quantities of the 36 volatiles compounds recorded for the individual reference plants together neatly separate the inbred lines, with the exception of line F7 and F113, which are only separated along the third axis (Fig. 6A). Hence the individual plants of a line form nice clusters, and an analogous PCA based on the average values per line revealed very similar relationships. The following parameters were found to significantly correlate with the factor scores of the six lines obtained by this latter PCA: mean caterpillar weight was negatively correlated with the scores of axis 1 (eigenvalue = 0.4043; *r* = −0.863, *p* = 0.024), and the number of spiders was positively correlated with the scores of axis 2 (eigenvalue = 0.314; *r* = 0.895, *p* = 0.012). Figure 6B provides a visual representation of the relationships among the various parameters, whereby the individual volatiles compounds were pooled (cumulative sum of the amounts) into groups of common biosynthetic origin. The measures of larval performance and the proportion of unparasitized caterpillars are negatively correlated with most volatile groups, as indicated by the fact that the respective factor loadings point in opposite directions.

## Discussion

The results obtained from collections of HIPVs under laboratory conditions seem to be globally representative for what can be found in the field, as suggested by the generally good match for many single compounds and for the cumulative amount of all volatiles (Fig. 5). However, we also observed some clear disparities regarding the relative proportions of some compounds in the odour blend. These differences concerned, above all, aromatic volatiles derived from the shikimic acid pathway [34], namely indole, benzyl acetate, phenethyl acetate and methyl anthranilate, which were underrepresented in the field, but not methyl salicylate, for which the opposite was true (Table 1). This discrepancy may likely be explained by the different biotic and abiotic environments, but could also be due to methodical problems met in the field. For example, condensation of water due to the high relative humidity inside the plastic bags may have altered the absorptive qualities of the Super-Q filters for certain compounds. Yet, this argumentation should not apply to sesquiterpenes, some of which were dominant in the field, but almost absent in the laboratory, and which - being isomers - share common chemical properties with the remaining sesquiterpenes. In this case, an alternative explanation for the difference between laboratory and field may be different degrees of plant maturation. At time of collection, the maize plants in the field were 33–49 days old, whereas the seedlings in the laboratory were only 9–12 days old. Köllner *et al.* [35] documented significant changes in sesquiterpene hydrocarbon content during maize development. Gouinguené [36] also reported age-specific variation in the proportion of HIPVs of maize plants in a laboratory assay, but the release of neither indole nor sesquiterpenes appear to be particularly affected by plant age classes ranging from 2 to 8 weeks. Moreover, the plants were fed upon by two different herbivores, *Spodoptera frugiperda* (field) and *Spodoptera littoralis* (laboratory), which may have further contributed to the observed divergence in volatile profiles [4], [37].

For logistical reasons, i.e. constraints in the equipment and manpower, we did not conduct the volatile collections directly on the numerous plants that were used for assessing performance and parasitism, which would have been desirable in the ideal situation, but on a separate smaller set of plants grown on an adjacent plot. The fact that the parasitism rate observed on an individual plant cannot be related to the volatiles emitted of that very same plant may be considered a limitation of our experimental design. Yet, since the reference plants used for odour collections were infested at the same time as the plants in the experimental plots, grew only a few meters away in the same field, and did not seem to differ regarding caterpillar performance, the data on volatiles can be considered as fairly representative for the whole set of plants.

We found considerable differences among the inbred lines both in survival (losses due to mortality and dispersal) and in the growth of *S. frugiperda*. Adult size, developmental time and growth rate are closely interrelated traits: on suboptimal foods, prolonged development can be viewed as means of increasing food intake to reach the same final weight as on higher quality foods that allow faster growth [38]. This is exemplified by larvae of the African armyworm *Spodoptera exempta*, who even use supernumerary moults to achieve final body size under poor conditions [39].

The differences in average weights observed in our field study cannot be explained by the factor leaf toughness. Indeed line F7, which had the toughest leaves, sustained the highest growth rates, the positive relationship being close to significant (Table 3). This counterintuitive result contradicts findings from studies on the European corn borer [31] and may be the consequence of trade-offs between the resource allocation for phenolic fortification of cell walls and direct chemical defences. In contrast, there was a clear negative correlation between average weights attained by the caterpillars and the volatile emission by the corresponding inbred lines. This nicely matches the results of a recent laboratory study covering a different set of 20 maize inbred lines: aboveground resistance against insects was positively correlated with the plant’s capacity to produce volatiles in response to insect attack [40]. The difference between the lowest and the highest average larval weight attained was about twofold in that study and hence roughly comparable with our findings. It is difficult to conceive that the HIPVs themselves exert a harmful impact on larval growth. The correlation may rather be explained by the shared jasmonic acid-dependent signalling cascade between HIPVs and metabolites that play a role in direct defence [41].

The pronounced variation in the performance of the caterpillars complicates the interpretation of the encountered differences in the parasitism rates among the lines. For *C. marginiventris*, these rates tended to increase slightly with the number of host larvae available on the plants in four out of the six lines, whereas no such density dependence was found for *C. sonorensis*. Therefore the variable caterpillar density is unlikely to be an important factor accounting for the differences in parasitism that we observed among the lines.

The slow-growth–high-mortality hypothesis predicts that prolonged development in herbivorous insects results in greater exposure to natural enemies. For example, fast-developing larvae of *Pieris rapae* incur less parasitism by *Cotesia glomerata* than slow-developing larvae [42]. We cannot rule out that this mechanism was operative also in our study, but its effect must have been very limited because we collected the caterpillars back, when most of them were still in a vulnerable state. Furthermore, the hypothesis cannot explain the big difference in parasitism rates between the two lines that allowed similar growth rates, F7 and W401. The same argument applies for the possibility that caterpillars growing on less suitable host plants suffer from a weakened immune system making them more susceptible to successful parasitization [43], [44].

This leaves us with our initial hypothesis, i.e. differential attraction of the parasitoids mediated by HIPVs. The cumulative quantity of all volatiles cannot account for the variation in parasitism: the highest rate was found on a low emitter and the second highest on a high emitter (Fig. 3). Out of the blend, only two volatile compounds qualified as potential key compound affecting host-location: (*E*)-2-hexenal correlated negatively with parasitism by *C. sonorensis* and methyl salicylate correlated positively with parasitism by *C. marginiventris*. Since this is probably what can be expected by chance alone, when testing 36 compounds at a significance level of 5%, these findings have to be interpreted with utmost caution. (*E*)-2-hexenal was detected only in comparatively small amounts, close to detection threshold and did not differ significantly among the lines. It has been identified as an attractant for carnivorous insects [45], but we are not aware of any study reporting a repellent effect of this green leaf odour for organisms of the third trophic level. Therefore we are rather inclined to consider this correlation simply a “random artefact”. The positive correlation with methyl salicylate is largely dependent on the “outlier” line W401, which produces nearly twice as much of this constituent as the other lines. Although the evidence that methyl salicylate plays a role in the foraging activity of *C. marginiventris* is not overly compelling, it is interesting to note that this compound is also suspected to play a role in attracting two other congeneric parasitoids, *Cotesia glomerata* and *Cotesia rubecula* to their *Pieris* hosts [18]. Methyl salicylate has also been shown to attract other natural enemies belonging to various taxonomic groups [46], such as lacewings [17] or predatory mites [47]. Since it is usually only released in trace amounts by maize seedlings, methyl salicylate may have escaped attention in previous laboratory studies. Yet, it readily elicits antennal responses in female *C. marginiventris* [48], and has been putatively associated with the high attractiveness of cowpea HIPVs for this wasp species [49]. Neither the overall amount of volatiles nor the amount of every individual compound represent necessarily the information exploited by the parasitoids. Yet, the differences in parasitism among the lines were not correlated with the factor loadings of a PCA of the volatiles, either. This may not be surprising in the case of axis 1, which explained about 40% of the variation, but separated the lines mainly according to total emission.

Since the absorbents used for odour collection possess a certain selectivity, some behaviourally active compounds may not have been screened with our method. Alternatively, they may have been present in the blend in quantities below detection threshold only. Our attempts to link differential parasitism to the emission of HIPVs by maize are hampered by a lack of knowledge about which components in the odour bouquet are responsible for the attraction of parasitoids [50]. Despite the fact that several extensive studies have been conducted on this topic, such key compounds remain to be identified, but the range of potential candidates has been narrowed down. Green leaf volatiles or further compounds that are instantaneously released upon damage are important attractants of inexperienced *C. marginiventris* [51]. Some common volatiles derived from shikimic acid, notably indole, appear not to be involved in the attraction of naïve *C. marginiventris* to host infested maize plants [52]. The sesquiterpenes, which make up a major part of the volatile blend both in number and quantity, apparently are also not essential for the initial attraction of *C. marginiventris* [49]. In contrast, a few as yet unidentified rather polar compounds are assumed to be behaviourally active at very low doses [49], [50].

The released females of *C. sonorensis* were found to be about two and a half times more efficient in parasitizing the caterpillars than those of *C. marginiventris*, but it should be noted that in the case of multiparasitism (i.e. more than one parasitoid species in one host) *C. sonorensis* almost always outcompetes *C. marginiventris* [53]. Therefore the number of hosts that were at one point parasitized by *C. marginiventris* is likely higher than what can be determined from the parasitoid emergence data. The parasitism rates observed on the six lines exhibited a similar pattern for both wasp species. In olfactometer tests naïve females of *C. sonorensis* and *C. marginiventris* also shared almost identical preferences for induced volatiles from different plant species. Yet, for *C. marginiventris* these preferences are strongly changed when wasps are given an oviposition experience on a specific plant, whereas no such behavioural modification was found in the choices of *C. sonorensis* [53]. Evidence for differential attractiveness has also been obtained in extensive olfactometer experiments with 18 maize inbred lines including the six lines chosen for the present study (Cristina Tamò, unpublished data) and with 8 maize varieties [54], respectively. In both cases, in congruence with the results from the current study, the recorded preferences were not correlated with the total amount of volatiles emitted.

From an agronomic point of view, the number of unparasitized *S. frugiperda* caterpillars remaining on the plant is the most important parameter, as it will determine the prospective damage. It differed almost threefold between the two extreme lines F7 and F476 (see N in Fig. 3I). HIPVs and correlates thereof might have accounted for a considerable part of this variation, with direct chemical defence and indirect defence through recruitment of beneficial insects apparently acting in union. The contribution of parasitism may indeed have been underestimated in this case, because we removed most of the caterpillars in a stage that was still susceptible to parasitoids. While our results are promising, many open questions need to be addressed, before we can judge whether enhancing the attractiveness of crop plants to beneficial insects is a realistic strategy for pest control in maize [25], [55], [56]. Further progress is needed in our efforts to identify the physiologically and behaviourally active factors in order to determine which plant traits to select for. Instead of correlative approaches that entirely rely on naturally available variation, mutants and transgenic plants with modified volatile emissions may prove a more powerful means of testing the ecological function as well as the application potential of HIPVs [45]. Moreover, there is a need for field studies at larger, ecologically and agriculturally relevant scales in order to eventually reveal the role of plant volatiles in determining the population dynamics of pests and their natural enemies [57].
